# Patient Expectations and Dentists’ Perceptions of Those Expectations in Dental Implant Therapy: A Mirror-Design Study

**DOI:** 10.3390/healthcare14152268

**Published:** 2026-07-24

**Authors:** Akın Özdemir, Mehmet Altay Sevimay, Ercan Karakaş

**Affiliations:** 1 Independent Researcher, 34680 Istanbul, Türkiye; 2 Department of Oral and Maxillofacial Surgery, Faculty of Dentistry, Gazi University, 06490 Ankara, Türkiye; altay97sevimay@gmail.com; 3 Department of Oral and Maxillofacial Surgery, Faculty of Dentistry, Eskişehir Osmangazi University, 26040 Eskişehir, Türkiye; ercan.karakas@ogu.edu.tr

**Keywords:** oral healthcare quality, expectation management, mirror-design questionnaire, patient-reported outcome measures, dental implant

## Abstract

Background/Objectives: Oral healthcare succeeds not only when treatment is biologically sound but when its outcome meets patient expectations, making expectation management a measurable component of care quality. Patient satisfaction in dental implant therapy is strongly influenced by pre-treatment expectations, yet differences between patient expectations and dentists’ perceptions of those expectations remain poorly characterized. This study compared the two using a mirror-design survey. Methods: A cross-sectional survey was administered to adult patients (*n* = 250) and dentists involved in implant therapy (*n* = 200). A 16-item questionnaire was developed in two parallel versions covering two domains, outcome expectations and surgical process apprehension, rated on a 5-point Likert scale. Internal consistency, item- and subscale-level differences, and signed and absolute between-group difference scores were analyzed. Results: Significant between-group differences were identified in 13 of the 16 items, while total scale scores did not differ between groups, producing a cancel-out effect at the aggregate level. Within the surgical process apprehension domain, patients were more optimistic than dentists perceived, with procedural pain showing the largest between-group difference (Cliff’s δ = 0.437). The opposite pattern emerged for long-term outcomes, where concern about implant loss yielded the largest difference across the entire instrument (δ = 0.645). Dentists’ clinical experience was negatively correlated with the signed total gap score (ρ = −0.342, *p* < 0.001), indicating reduced directional bias among more experienced clinicians. Conclusion: Patients’ expectations differed systematically from dentists’ perceptions of those expectations across both domains, but these opposing differences offset at the aggregate level. Aggregate summary scores may therefore obscure clinically relevant item-level differences, and directional item-level analyses may better capture expectation patterns in implant dentistry.

## 1. Introduction

The goal of oral healthcare extends beyond biological and technical success to the patient’s own perception of that success, which is shaped in large part by pre-treatment expectations. Contemporary healthcare quality frameworks emphasize patient-centered care, defined by responsiveness to patients’ preferences, needs, and values, as a core dimension of high-quality care [1]. Patient experience is also closely linked to clinical effectiveness and patient safety, supporting the view that subjective patient perspectives should not be treated as secondary outcomes [2]. Accurate clinician understanding of these expectations is therefore increasingly recognized as an essential component of care quality; however, clinicians across medical and dental disciplines frequently misjudge their patients’ concerns, health beliefs, and priorities [3,4]. A clinically successful outcome that fails to meet patient expectations does not fully achieve the aims of care; consequently, the alignment between what patients expect and what clinicians believe they expect is itself a quality concern. Dental implant therapy—a high-stakes, high-investment, and expectation-sensitive intervention within oral healthcare—offers a particularly informative setting in which to examine this group-level difference between patients’ and clinicians’ expectations [5].

Dental implant therapy has become a standard treatment modality for the rehabilitation of partially or completely edentulous patients, with millions of implants placed annually worldwide. Clinical research has traditionally focused on objective indicators of success such as implant survival, marginal bone stability, and absence of biological or technical complications [6], with 10-year survival rates exceeding 90% across long-term studies and systematic reviews [7,8,9]. Despite this clinical predictability, treatment success defined solely by such biological and technical criteria may not capture the full picture of how patients themselves evaluate the outcomes of implant therapy. In recent years, a paradigm shift has emerged in implant dentistry toward patient-centered evaluation of treatment success. Within this framework, patient-reported outcome measures (PROMs) have become essential tools for capturing subjective perspectives on function, aesthetics, and psychosocial well-being. Patient satisfaction is widely recognized as a key indicator of healthcare quality and in implant dentistry high satisfaction rates have been consistently reported, particularly regarding chewing ability, comfort and aesthetic appearance. Nevertheless, satisfaction is strongly shaped by pre-treatment expectations: patients frequently approach implant therapy anticipating outcomes comparable to natural dentition and prior investigations have demonstrated that such expectations are often high and directly influence perceived treatment success [5,9].

Understanding patient expectations has become a central component of implant treatment planning because unrealistic expectations can preclude patient satisfaction even when the clinical outcome is objectively successful. Dentists, on the other hand, have their own way of evaluating implant therapies, and they base these evaluations on biological, technical, and prognostic factors. This has been shown in earlier studies to create discrepancies between patient expectations and dentists’ perceptions in terms of patient satisfaction and success [10,11].

Beyond the field of implantology, the broader literature on patient–dentist concordance has consistently shown that clinicians often misjudge the experiences and concerns of their patients. In a large investigation of 197 practitioners and 5879 patients within the National Dental Practice-Based Research Network, dentists frequently failed to recognize patient dissatisfaction with restorative treatment, particularly with regard to the patient’s desire for greater communication [12]. More recently, a mirror-design questionnaire study among 1121 patients and 77 dentists in 41 dental practices identified specific items where patient experiences and dentist perceptions diverged in the context of treatment satisfaction and loyalty [13]. Validated instruments developed for assessing dentists’ self-perception in oral healthcare management have likewise emphasized the value of structured questionnaires for capturing professional viewpoints on care delivery [14]. However, the majority of these studies have focused on post-treatment satisfaction with restorative or general dental care rather than on pre-treatment expectations specific to implant therapy. Implant therapy is a high-stakes, high-investment intervention in which expectation management is central to perceived treatment success.

Although patient satisfaction and patient-reported outcomes have been extensively investigated in implant dentistry, comparatively few studies have systematically examined the alignment between what patients expect from implant therapy and what dentists perceive those expectations to be. To our knowledge, no previous study has applied a mirror-design methodology specifically to pre-treatment expectations in dental implant therapy, combined with item-level between-group comparisons and effect size quantification.

Accordingly, the present study aimed to (i) develop and psychometrically assess a 16-item mirror-design instrument assessing expectations regarding implant therapy from both patient and dentist perspectives; (ii) quantify the magnitude and direction of between-group differences at the item level and characterize subscale-level expectation patterns within each group; and (iii) examine whether directional difference scores varied according to demographic and professional characteristics, with particular attention paid to dentists’ clinical experience. We hypothesised that patients and dentists would differ systematically in their expectations across distinct content domains, with the magnitude and directionality of disagreement varying by domain and by dentist clinical experience. Findings from this study may inform pre-treatment counseling strategies, support communication training in implant dentistry, and contribute to greater alignment between patient expectations and realistic clinical outcomes.

## 2. Materials and Methods

### 2.1. Study Design and Ethical Approval

This cross-sectional, comparative, questionnaire-based study employed a mirror-design methodology to assess patients’ expectations regarding dental implant therapy and in parallel, dentists’ perceptions of those expectations. The mirror-design (parallel-version) approach was selected because the research question concerns two perspectives on a single construct: what patients expect from implant therapy, and what dentists believe patients expect. Answering it requires both groups to respond to semantically identical content on an identical metric, so that any between-group difference reflects perspective rather than item wording, coverage, or response format. Each item was therefore prepared in two versions differing only in the grammatical person of the respondent (first person for patients, third person for dentists), with the construct, item order, and five-point Likert scale held constant. Independently developed patient and clinician instruments are not comparable at the item level, and post hoc alignment of non-parallel items would confound perspective with measurement. Because the cohorts were sampled independently rather than as matched dyads, the design supports group-level comparison rather than within-pair agreement; item-level effect sizes (Cliff’s δ), not dyadic agreement statistics, were therefore appropriate.

The study was conducted at the Faculty of Dentistry, Gazi University. Ethical approval was obtained from the Clinical Research Ethics Committee of Gazi University (Approval No.: 2026-574, Date: 30 March 2026). The study adhered to the Declaration of Helsinki, and reporting followed the STROBE guidelines for cross-sectional studies. Written informed consent was obtained from all patient participants prior to questionnaire administration; for dentists, electronic informed consent was obtained at the start of the online survey, prior to access to the questionnaire items.

Because patients and dentists were recruited as two independent samples rather than as matched patient–dentist dyads, all comparisons reported in this study reflect group-level differences between patients’ expectations and dentists’ perceptions of those expectations rather than agreement within individual patient–dentist pairs.

### 2.2. Participants

Eligible patients were adults (≥18 years) presenting at the Faculty of Dentistry, Gazi University, for initial dental implant consultation who had not yet received implant therapy and were able to comprehend the questionnaire in Turkish. Patients with prior implant experience, severe systemic contraindications or cognitive conditions precluding self-report were excluded. Patients were recruited through consecutive sampling. Of 322 patients invited, 72 declined, yielding a final patient sample of 250 (response rate 77.64%).

**Figure 1:**
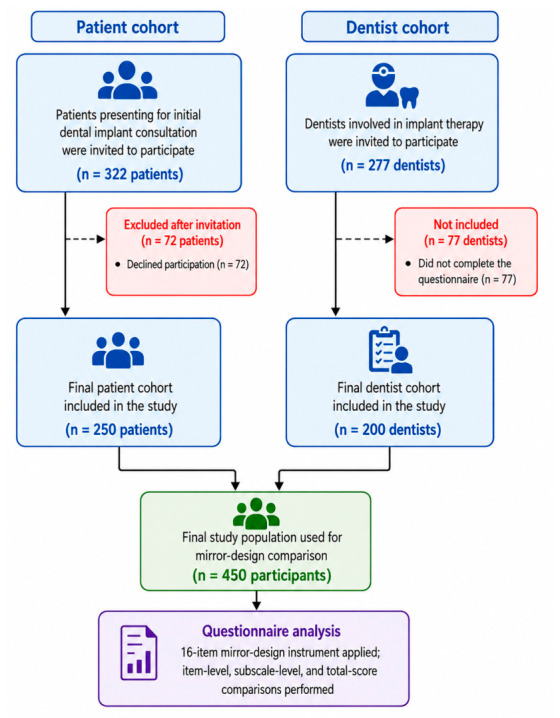
Participant flow diagram. The diagram summarizes the recruitment, exclusion, inclusion, and final analysis process for the patient and dentist cohorts.

Dentists were practitioners actively involved in implant therapy (surgical placement, prosthetic rehabilitation, or both). They were recruited through convenience sampling. A personalized survey invitation containing a unique link to the online questionnaire was sent directly to 277 dentists across the relevant specialties (Oral and Maxillofacial Surgery, Periodontology, Prosthodontics, general practice, and other specialties) to ensure representation. Because invitations were sent to a defined list of individually identified dentists rather than through open circulation, the number of invitees could be determined precisely. Of the 277 dentists invited, 200 completed the questionnaire, corresponding to a response rate of 72.2%. The participant recruitment and inclusion process for both cohorts is summarized in Figure 1.

### 2.3. Sample Size

The target sample size was determined a priori. For exploratory factor analysis, both samples met the 10:1 subject-to-item ratio recommended by Nunnally [15]. For Cronbach’s α, sample sizes ≥ 200 yield 95% confidence interval half-widths ≤ 0.05. An a priori power analysis in G*Power 3.1 indicated a minimum of 105 participants per group to detect a medium between-group effect (Cohen’s d = 0.50) at α = 0.05 with power = 0.95 [16]; the achieved samples exceeded this requirement.

### 2.4. Instrument

A 16-item instrument was developed through a structured three-stage process. First, an initial pool of candidate items was developed through a literature-informed item-generation process, guided by previously published instruments on patient expectations in implant dentistry [5,12,13] and organized around two conceptual domains: outcome expectations (aesthetic, functional and long-term success-related perceptions) and surgical process apprehension (perioperative and psychological concerns).

Second, content validity was assessed by a panel of eight expert clinicians (specialists in periodontology, oral and maxillofacial surgery, and prosthodontics), each with at least five years of clinical experience in implant therapy. The patient and dentist versions were reviewed in two separate rounds. Each item was rated for relevance on a four-point scale (1 = not relevant to 4 = highly relevant) [17]. Of the initial pool of 21 candidate items, 5 items with item-level CVI below 0.78 were eliminated and 2 items were rephrased to improve clarity following expert feedback; rephrased items were re-evaluated in a subsequent review round. The retained 16-item instrument yielded a scale-level CVI (S-CVI/Ave) of 0.88, indicating good content validity [18]. Third, the revised 16-item instrument was pilot-tested on 20 patients and 20 dentists who were not included in the final analysis; the pilot confirmed item clarity and acceptable preliminary internal consistency, and no further revisions were required. The full wording of all 16 mirror-design items in both the patient and clinician versions is presented in Table 1, allowing direct comparison of the semantically equivalent paired statements used to evaluate differences between the two groups.

Two parallel versions were prepared: a patient version (e.g., “I believe that dental implants will restore my chewing function”) and a dentist version (e.g., “Patients believe that dental implants will restore their chewing function”). Both versions contained semantically equivalent paired items addressing the same 16 expectation constructs, with phrasing adapted only to reflect the respondent’s perspective (first-person for patients; third-person for dentists). Items were scored on a five-point Likert scale (1 = strongly disagree to 5 = strongly agree). One item was phrased in the negative direction in the patient version (item M11, addressing anxiety about the surgical procedure) and was reverse-coded before analysis (new score = 6 − raw score); the mirrored dentist-version item was worded in the positive direction and required no recoding, so that higher scores consistently reflected more positive expectations across both versions. Sociodemographic information (age, sex, and education level for patients; age, sex, professional experience, and specialty for dentists) was also collected. Because the two versions were mirrored, the 16 expectation items were held parallel across groups, whereas background variables were tailored to each role. For patients, age, sex, and educational level were recorded as established correlates of implant-related knowledge and dental anxiety; age was also examined as a source of variation in expectations in the subgroup analyses (Section 3.5.1), where it was significantly associated with expectation scores. For dentists, age, sex, years of experience, and specialty were recorded as the professional characteristics most likely to shape perceptions of patient expectations. 

### 2.5. Data Collection

Instrument development—comprising the expert content-validity panel, item revision, and pilot testing—was conducted between 1 and 10 April 2026, following ethical approval on 30 March 2026. The main survey data were subsequently collected from patients and dentists beginning 10 April 2026. Patient questionnaires were self-administered in a private setting at the Faculty of Dentistry, Gazi University, with a trained research assistant available to clarify procedural questions but not to interpret item content. Patients completed the questionnaire before receiving any detailed treatment-related information, in order to capture expectations uninfluenced by consultation content. Dentist questionnaires were administered electronically through Google Forms (Google LLC, Mountain View, CA, USA). Each invited dentist received a personal invitation containing the survey link; the survey was configured to accept only one response per Google account to limit duplicate submissions. Responses were collected anonymously, with no personally identifiable information recorded. Each item required a response before questionnaire submission; consequently, the final dataset contained no missing values. Data were double-entered and cross-verified by two independent researchers.

### 2.6. Statistical Analysis

All statistical analyses were performed using IBM SPSS Statistics v.27 (SPSS Inc., Chicago, IL, USA). Statistical significance was set at α = 0.05 (two-tailed). Continuous variables were summarized as means and standard deviations (SD), together with medians and interquartile ranges (IQR); categorical variables were summarized as frequencies and percentages. The assumption of normality was assessed using the Shapiro–Wilk test, and homogeneity of variance was assessed using Levene’s test.

The suitability of the data for exploratory factor analysis was evaluated using the Kaiser–Meyer–Olkin (KMO) measure of sampling adequacy and Bartlett’s test of sphericity. Principal Axis Factoring (PAF) with Varimax rotation was applied for factor extraction, with the number of retained factors determined by Kaiser’s eigenvalue > 1 criterion in conjunction with scree-plot inspection. Factor analysis was performed separately for the patient and dentist samples. Internal consistency was evaluated using Cronbach’s α for the total scale and for each subscale; values ≥ 0.70 were considered acceptable [15].

For comparisons between two independent groups, an independent-samples *t*-test was used when assumptions of normality and homogeneity of variance were satisfied; otherwise, the Mann–Whitney U test was applied. For comparisons among three or more independent groups, one-way analysis of variance (ANOVA) was used when assumptions were satisfied; otherwise, the Kruskal–Wallis test was applied. Post hoc pairwise comparisons following significant ANOVA or Kruskal–Wallis results were performed using the Bonferroni correction. To control the Type I error rate across item-level comparisons between patients and dentists, the Benjamini–Hochberg false discovery rate (FDR) correction was additionally applied. Effect sizes for between-group comparisons were reported as Cliff’s δ, with thresholds of |δ| < 0.147 (negligible), 0.147–0.33 (small), 0.33–0.474 (medium), and ≥0.474 (large) [19].

F1 and F2 subscale scores were calculated as the sum of the items loading on each respective factor. For each item, a signed gap score was computed as the difference between the patient mean and the dentist mean (Mean_patient − Mean_dentist), preserving the direction of the between-group difference, whereas an absolute gap score was computed as the absolute value of this difference. The mean absolute gap across the 16 items was calculated as a global index of between-group difference. Subscale-level gap scores were derived analogously as the difference between patient and dentist mean subscale scores. For the subgroup analyses (Section 3.5.1 and Section 3.5.2) and the correlations with dentists’ clinical experience, gap scores were additionally computed at the respondent level, defined for each participant as the mean across the 16 items of the difference between that participant’s item score and the opposite group’s mean score on the same item, with signed and absolute versions derived as above; the group-level item gaps defined above were used only for the global mean-absolute-gap index. Spearman’s rank-order correlation coefficient (ρ) was used to examine the associations between dentists’ years of clinical experience and the dentist questionnaire scores (total and subscale) as well as the patient–dentist gap scores (both signed and absolute, at both total and subscale levels).

## 3. Results

### 3.1. Study Population

A total of 250 patients (132 female, 118 male; modal age group 45–54 years) and 200 dentists (111 female, 89 male) participated. The patient cohort was predominantly bachelor’s-degree-educated (46.8%). The dentist cohort was markedly younger, with 51.0% aged ≤34 years; 38.0% had 0–5 years of clinical experience and 35.5% had ≥11 years. Most dentists reported a specialty (74.0%): periodontology (21.0%), oral and maxillofacial surgery (19.5%), and prosthodontics (19.0%). Full demographic details are presented in Table 2.

### 3.2. Structural Validity and Reliability of the Questionnaire

Both questionnaires met the criteria for factor analysis (KMO: 0.883 for patients and 0.887 for dentists; Bartlett’s *p* < 0.001 for both; Table 3). Principal axis factoring with Varimax rotation identified a two-factor solution in each sample, explaining 37.7% and 41.3% of the total variance for patients and dentists, respectively. The two-factor solution was supported by both the Kaiser criterion (eigenvalues > 1) and visual inspection of the scree plot (Supplementary Figure S1). The two factors corresponded to the a priori domains: Outcome Expectations (F1) and Surgical Process Apprehension (F2). Notably, items 15 and 16 loaded onto F2 in the patient sample but onto F1 in the dentist sample, suggesting that patients frame long-term maintenance concerns as part of procedural apprehension, whereas dentists conceptualize them as outcome indicators. Internal consistency was acceptable across both samples: Cronbach’s α ranged from 0.762 to 0.873 for patients and from 0.724 to 0.886 for dentists (all > 0.70; Table 3).

### 3.3. Item-Level Comparison Between Patients and Dentists

Statistically significant differences were observed in 13 of the 16 items after Benjamini–Hochberg correction (Table 4); M1, M6, and M15 did not reach significance (all FDR-*p* > 0.05).

Between-group differences were bidirectional and followed a domain-specific pattern. Within the surgical process apprehension domain, patients reported significantly higher scores than dentists anticipated on all five significant items (M7–M11), with the largest effects for procedural pain (M7; δ = 0.437) and psychological tolerance (M11; δ = 0.338). Patients also rated two esthetic items (M2, M3) higher than dentists expected.

In contrast, dentists systematically overestimated patient expectations across the functional and long-term outcome items (M4, M5, M12–M14, M16). The largest between-group difference observed in the instrument was observed for M16 (implant loss concern): dentists anticipated a mean of 3.75, whereas patients themselves reported 2.42 (δ = 0.645, large), indicating that dentists substantially underestimated patients’ anxiety about long-term implant retention.

Collectively, these findings reveal that patients were more optimistic than dentists assumed regarding the surgical experience, while dentists assumed patients would be more concerned than patients actually were regarding long-term outcomes. Despite these systematic shifts, median scores in both groups remained within the central Likert range (2–4), reflecting differences in magnitude rather than extreme polarization.

### 3.4. Total Score Comparison Between Patients and Dentists

In contrast to the pronounced item-level differences described above, no statistically significant difference was observed between patients and dentists in the total questionnaire score (Table 5). The mean absolute gap across the 16 items was 0.36 Likert-scale points, indicating only modest overall between-group differences at the global level.

This pattern reflects a cancel-out effect at the scale level: although patients and dentists differed significantly on 13 of the 16 individual items, the items on which patients scored higher (within the surgical process apprehension domain) were numerically counterbalanced by the items on which dentists scored higher (within the long-term outcome expectations sub-domain). When summed across all items, the opposing directional gaps largely offset one another, yielding nearly equivalent total scores in the two groups. This finding underscores the value of item-level analyses when comparing patient expectations with dentists’ perceptions, as aggregate scores suggesting little overall difference may conceal clinically meaningful item-level differences.

### 3.5. Subgroup Analyses

These subgroup analyses address the study’s third aim of characterizing how expectations (in patients) and perceptions (in dentists) vary with demographic and professional characteristics, thereby identifying subgroups for whom pre-treatment communication may most need tailoring. Within-group subgroup analyses were performed separately for the patient and dentist cohorts. Because the empirically derived F1 and F2 subscales comprised different numbers of items in the two samples, subscale scores were compared only within each cohort and were not contrasted between patients and dentists.

#### 3.5.1. Patient Subgroup Analyses

Within the patient cohort, expectation scores varied significantly by age, sex, and educational level (Table 6). Across age groups, total scale scores (*p* = 0.002) and F1 scores (*p* < 0.001) differed significantly, with patients aged 45–54 years reporting higher F1 scores than each of the three younger age groups (Bonferroni-adjusted *p* < 0.05). No significant age-related differences were observed for F2 scores (*p* = 0.120) or for absolute gap scores (*p* = 0.250).

By sex, male patients reported significantly higher scores than female patients on the total scale (*p* < 0.001), F1 (*p* = 0.022), and F2 (*p* = 0.002). Absolute gap scores did not differ by sex (*p* = 0.324). Patients with primary/middle-school or high-school education reported higher total scale, F1, and F2 scores than those with a bachelor’s degree or postgraduate education (all *p* < 0.001; Bonferroni-adjusted). Absolute gap scores did not differ by educational level (*p* = 0.449).

#### 3.5.2. Dentist Subgroup Analyses

Within the dentist cohort, total scale and F1 scores varied substantially by age, clinical experience, and specialty, whereas F2 scores were stable across age and clinical experience but differed by specialty, and absolute gap scores were stable across all subgroups (Table 6).

Total scale and F1 scores decreased progressively with age (both *p* < 0.001): dentists aged ≤34 years reported markedly higher scores than those aged 35 years or older, with pairwise differences reaching *p* < 0.001. The same pattern was observed for clinical experience: dentists with 0–5 years of practice reported significantly larger total scale and F1 scores than those with 6–10 or ≥11 years of experience (both *p* < 0.001). Neither age nor clinical experience was significantly associated with F2 scores or absolute gap scores. All pairwise comparisons were Bonferroni-adjusted.

Specialty was significantly associated with total scale, F1, and F2 scores (all *p* < 0.001). Periodontists and prosthodontists reported lower total scale and F1 scores than oral and maxillofacial surgeons, general practitioners, and dentists of other specialties (Bonferroni-adjusted *p* < 0.001 for the relevant pairwise contrasts). Sex was not associated with any of the four scores (all *p* > 0.10).

#### 3.5.3. Association Between Dentist Experience and Between-Group Difference Scores

Dentist age and years of clinical experience were strongly collinear in this sample; age is therefore reported as a descriptive characteristic only, and the directional-bias findings reported below are framed in terms of clinical experience rather than age. Spearman’s rank-order correlation analyses examined the relationship between dentists’ years of clinical experience and the directional difference scores. Clinical experience was not significantly associated with the absolute gap score at either the total scale or the subscale level (all |ρ| ≤ 0.024; all *p* > 0.05).

However, clinical experience was significantly and negatively associated with the signed total gap score (ρ = −0.342, *p* < 0.001) and the signed F1 gap score (ρ = −0.403, *p* < 0.001) but not with the signed F2 gap score (ρ = −0.065, *p* = 0.357). At the group level, this means that the estimates of more experienced clinicians deviated less, directionally, from patient means.

## 4. Discussion

### 4.1. Overview of Principal Findings

This study compared patients’ pre-treatment expectations with dentists’ perceptions of those expectations before dental implant therapy. Item-level divergence was the rule rather than the exception, despite equivalent total scale scores. The signed gap between expectation and perception also narrowed as clinical experience increased, indicating a systematic, experience-related bias rather than random noise. These findings extend previous research on differences between patient expectations and clinicians’ perceptions in dental care [12,13] to the specific context of pre-treatment expectations in implant dentistry. In the sections that follow, we first interpret each cohort on its own terms and then bring the two perspectives together, because the clinically important signal lies in the direction of the divergences, not merely in their presence.

### 4.2. Clinicians’ Perceptions of Patient Expectations

Dentists’ perceptions were structured by professional rather than demographic factors. Specialty and cumulative experience shaped total and outcome-expectation (F1) scores, while perceptions of surgical apprehension (F2) were comparatively insensitive to these factors: dentist age and clinical experience influenced total and F1 scores without affecting F2. Periodontists and prosthodontists returned more conservative estimates than oral and maxillofacial surgeons, general practitioners, and other specialists (all *p* < 0.001 for total), a pattern reflected in both F1 (*p* < 0.001) and F2 (*p* < 0.001) and consistent with a practice profile centred on peri-implant complications and long-term prosthetic maintenance [20,21]. Clinical experience showed a strong gradient on total and F1 scores (both *p* < 0.001) but no association with F2 (*p* = 0.341). The same dissociation appeared in the directional gap between perception and patient report: dentist experience was associated with the signed F1 gap (ρ = −0.403, *p* < 0.001) but not the signed F2 gap (ρ = −0.065, *p* = 0.357). Taken together, this domain-specific patterning suggests that clinicians’ estimates reflect the clinical exposure characteristic of their discipline rather than a uniform calibration effect, and that the experience-related correction operates specifically on long-term outcome expectations.

Two mechanisms may explain why clinicians tended to anticipate greater apprehension than patients reported. Dentists routinely manage perioperative complications and anxiety, and this background may anchor their professional outlook toward a worst-case framing. That the accuracy of outcome-expectation estimates improved with experience, while apprehension estimates did not, is consistent with clinicians’ well-documented tendency to misjudge patient perspectives [3,4,22,23], an effect that appears to attenuate with clinical experience.

### 4.3. Patients’ Expectations

Patients’ expectations, by contrast, were structured by demographic characteristics. Male patients reported higher total scores than female patients (54.81 vs. 51.68, *p* < 0.001), reflecting higher scores on both F1 (*p* = 0.022) and F2 (*p* = 0.002); the lower female F2 scores, indicating greater pre-operative apprehension, are consistent with the well-documented sex gap in dental fear and anxiety [24,25,26]. An educational gradient was also evident—patients with primary or high-school education reported higher total, F1, and F2 scores than those with bachelor’s or postgraduate degrees (all *p* < 0.001), most strongly on F1—in line with evidence that education predicts implant-related knowledge and decision-making [27] and that knowledge deficits regarding longevity, maintenance, and complications are most pronounced among individuals with limited formal education [28,29].

Patients also appeared to approach the procedure with an optimism not fully grounded in knowledge of perioperative risk. In a multicentre survey, a substantial proportion of pre-consultation patients endorsed the misconception that implant treatment carries no meaningful risks [30], and fear of pain, swelling, or surgery was reported as a discouraging factor by only about one in ten periodontal patients [31]; such apprehension is nonetheless prevalent and largely independent of age [32,33]. A parallel pattern may hold for long-term outcomes, where optimism may reflect gaps in pre-treatment information: in one cohort, only 58.9% of patients recalled being informed about possible complications despite 91.6% being told about periodic check-ups [34], and earlier surveys documented widespread beliefs that implants require no more care than natural teeth alongside high longevity expectations [35]. Because insufficient pre-treatment information predicts inferior satisfaction [36] and baseline knowledge varies widely between individuals [37], these expectation profiles are plausibly modifiable through counselling.

### 4.4. Comparing the Two Perspectives

Items 15 and 16 loaded onto different factors in the patient and dentist samples; the F1/F2 domain scores are therefore not strictly equivalent across groups, and the following comparisons should be read as parallel descriptive patterns within each cohort rather than as strict cross-group equivalences. Placed side by side, the two cohorts diverged in opposite directions across the two domains. Within surgical-process apprehension, patients were consistently more optimistic than dentists assumed, most markedly for procedural pain (M7, Cliff’s δ = 0.437) and psychological tolerance of treatment (M11, δ = 0.338, reverse-coded). For long-term outcomes, the pattern reversed: dentists underestimated patient concern, with the largest divergence of the entire instrument observed for implant loss (M16, δ = 0.645; patient mean 2.42 vs. dentist mean 3.75). This asymmetry is what makes the findings actionable, because it identifies where pre-treatment counselling is most likely to misfire—over-addressing procedural fears that patients hold less strongly than clinicians assume, while under-addressing the long-term concerns patients actually carry.

A systematic divergence between patient and clinician judgments is well established in implant dentistry, although it has been documented mainly for post-treatment esthetic outcomes rather than pre-treatment expectations. Clinicians consistently apply more critical standards than patients, who rate the same restorations more favourably [38,39], and objective clinician indices frequently diverge from patients’ subjective appraisals [40]. Our findings show that this patient–clinician asymmetry is already present before treatment, at the level of expectations, and extend it across both the outcome-expectation and surgical-apprehension domains through a parallel mirror design, which to our knowledge is the first application of this approach to pre-treatment expectations in implant therapy.

The two domains also behaved differently across subgroups. Outcome-related expectations (total and F1) shifted substantially with patient demographics and dentist professional characteristics, whereas surgical apprehension (F2) followed a more selective pattern, remaining stable across age, professional experience, and clinician sex but differing with patient sex, patient educational level, and dentist specialty. Absolute gap scores, in contrast, were unaffected by any of the examined characteristics, indicating that while the direction of misperception varied systematically, its overall magnitude was broadly consistent across participants.

Critically, this signal was visible only at the item level. Because the opposing gaps offset one another, total scores suggested agreement, and a summary-score analysis would have concluded that patients and clinicians were well aligned—concealing precisely the misalignment that matters clinically. Directional, item-level analysis is therefore required to surface these gaps. This cancel-out effect is partly a consequence of combining favourable outcome items with apprehension-oriented items within a single scale, so it should be read as a feature of the present design rather than a general methodological principle.

Beyond their descriptive value, these directional mismatches carry practical implications for medico-legal risk. When patients’ pre-treatment expectations go unrecognized, the resulting gap between anticipated and experienced outcomes can fuel dissatisfaction and patient–clinician conflict, which underlie a substantial share of complaints and malpractice claims [41]. Such disputes are, in turn, a principal driver of defensive medical practice, in which decisions are shaped by legal self-protection rather than patient benefit [41,42]. Aligning pre-treatment communication with patients’ actual expectation profile may therefore serve not only care quality but also as a safeguard against complaints and the drift toward defensive practice.

### 4.5. Strengths

This study has several strengths, including the rigorously validated three-stage mirror-design instrument (Section 2.4). The analysis applied layered multiple-comparison correction (Benjamini–Hochberg false discovery rate and Bonferroni adjustment) together with effect-size quantification using Cliff’s δ, a non-parametric measure that does not depend on distributional assumptions or measurement invariance and is therefore well suited to ordinal, cross-cohort comparison.

### 4.6. Limitations

Several limitations should also be acknowledged. First, patients were recruited consecutively at a single academic centre, and dentists were recruited by convenience sampling, which may limit generalizability to other healthcare settings and regions. Accordingly, the reported expectation profiles should be regarded as provisional and confirmed in multi-centre samples before broader generalization. Second, a formal multi-group confirmatory factor analysis to establish measurement invariance between the patient and dentist cohorts was not performed. In mirror-design instruments, the aim is not necessarily to demonstrate strict latent equivalence between groups but rather to explore potential differences in how parallel items are conceptually organized and interpreted. This was reflected in the exploratory factor analysis, where items 15 and 16 loaded onto different factors across the two cohorts. Because the empirically derived factor structures were not fully identical, direct between-group comparisons at the subscale level were not considered methodologically appropriate. Accordingly, comparisons between patients and dentists were conducted primarily at the item level using Cliff’s δ, a non-parametric effect size measure that does not require measurement invariance assumptions, whereas subscale-level analyses were interpreted only within each cohort.

Third, several items were intentionally framed in absolute terms (e.g., implants being indistinguishable from natural teeth [M1], lasting a lifetime [M12], or remaining trouble-free without check-ups [M14]) to probe unrealistic expectations. Although such wording could elicit reaction-driven responses, it did not appear to inflate between-group differences: these items showed small or non-significant differences (M1, δ = 0.06, ns; M12, δ = 0.15; M14, δ = 0.19), whereas the largest between-group differences were observed for more moderately worded items (M16, δ = 0.65; M7, δ = 0.44). Fourth, the cross-sectional design captures expectations at a single time point and relies on self-report, which may be subject to social desirability and recall-related biases. Future longitudinal research linking pre-treatment expectations to post-treatment satisfaction, together with controlled trials of structured pre-treatment communication, would help establish whether reducing differences between patient expectations and clinicians’ perceptions translates into measurable improvements in patient-reported outcomes. Finally, patients and dentists were sampled as two separate groups rather than as matched patient–dentist pairs. This is a natural feature of the mirror-design approach, which compares expectation patterns at the group level rather than within individual pairs. Relatedly, patients responded before detailed consultation and dentists responded without specific case context, so the comparison reflects generalized population-level expectations and perceptions rather than expectations arising within an individual clinical encounter; patients’ prior exposure to implant information from other sources was also not assessed. Accordingly, all reported difference measures should be interpreted as reflecting group-level comparisons rather than agreement within patient–dentist pairs.

## 5. Conclusions

Patient expectations and dentists’ perceptions of those expectations before dental implant therapy differed systematically and followed distinct directional patterns across questionnaire items. Patients anticipated less procedural discomfort than dentists assumed, whereas dentists appeared to underestimate patients’ concerns regarding long-term outcomes, particularly implant loss. Because opposing directional gaps offset at the aggregate level, total scores may obscure clinically relevant item-level differences, and directional item-level analysis may therefore be more informative than aggregate summary scores alone.

From a healthcare perspective, the results highlight expectation management as an essential component of patient-centered implant care. Structured pre-treatment communication may need to address not only the surgical procedure but also long-term prognosis, maintenance requirements, possible complications, and implant loss. Integrating such evidence-based communication into routine implant counseling may help reduce patient–clinician asymmetry and support shared decision-making; however, these clinical implications should be interpreted as hypothesis-generating, as the cross-sectional design of the present study does not permit causal inference regarding improved patient outcomes, and confirmation in prospective and interventional studies is required.

## Figures and Tables

**Table 1:** Mirror-design questionnaire items used in the patient and clinician versions of the survey.

Item	Patient Version	Clinician Version
1	I believe that my implant-supported restoration will be indistinguishable from a natural tooth.	Patients expect implant-supported restorations to be indistinguishable from natural teeth.
2	I expect implant treatment to markedly improve the appearance of my smile.	Patients expect implant treatment to markedly improve the appearance of their smile.
3	I expect the gingival appearance around my implant to be flawless.	Patients expect the gingival appearance around their implant to be flawless.
4	I believe that my implant will fully restore my chewing ability.	Patients believe that implants will fully restore their chewing ability.
5	I expect my biting force after implant treatment to reach the same level as with my natural teeth.	Patients expect their biting force after implant treatment to reach the same level as with natural teeth.
6	I expect my implant to provide a balanced and harmonious occlusion with the opposing dentition.	Patients expect their implant to provide a balanced and harmonious occlusion with the opposing dentition.
7	I believe that implant surgery will not be very painful.	Patients believe that implant surgery will not be very painful.
8	I expect the healing process to be completed within a short period of time.	Patients expect the healing process to be completed within a short period of time.
9	I believe that implant treatment will not significantly interfere with my daily life.	Patients believe that implant treatment will not significantly interfere with their daily life.
10	I believe that the risk of complications following surgery will be low.	Patients believe that the risk of complications following surgery will be low.
11	I find implant treatment psychologically challenging. ^1^	Patients perceive implant treatment as a psychologically straightforward process.
12	I believe that my implant will last a lifetime.	Patients believe that their implant will last a lifetime.
13	I believe that the likelihood of implant treatment failure is very low.	Patients believe that the likelihood of implant treatment failure is very low.
14	I believe that my implant will remain trouble-free even without regular check-up appointments.	Patients believe that their implant will remain trouble-free even without regular check-up appointments.
15	I believe that maintaining my implant will not be difficult.	Patients believe that maintaining an implant is not difficult.
16	I am not concerned about the possibility of losing my implant.	Patients are not concerned about the possibility of implant loss.

^1^ Reverse-coded item; the patient version was negatively worded and recoded as 6 − raw score before analysis.

**Table 2:** Demographic and clinical characteristics of patients (*n* = 250) and dentists (*n* = 200).

	*n*	%
Patients (*n* = 250)		
**Age**		
≤25 years	37	14.8
26–34 years	43	17.2
35–44 years	49	19.6
45–54 years	52	20.8
55–64 years	51	20.4
≥65 years	18	7.2
**Sex**		
Female	132	52.8
Male	118	47.2
**Education Level**		
Primary–Middle School	35	14.0
High School	47	18.8
Bachelor’s Degree	117	46.8
Master’s/Doctorate	51	20.4
Total	250	100.0
**Dentists (*n* = 200)**		
**Age**		
≤25 years	40	20.0
26–34 years	62	31.0
35–44 years	39	19.5
45–54 years	30	15.0
55–64 years	26	13.0
≥65 years	3	1.5
**Sex**		
Female	111	55.5
Male	89	44.5
**Years of Professional Experience**		
0–5 years	76	38.0
6–10 years	53	26.5
≥11 years	71	35.5
**Specialty**		
General Practitioner	52	26.0
Periodontology	42	21.0
Oral & Maxillofacial Surgery	39	19.5
Prosthodontics	38	19.0
Other	29	14.5
Total	200	100.0

**Table 3:** Exploratory factor analysis results and internal consistency coefficients for the patient and dentist questionnaires.

	Patient Questionnaire		Dentist Questionnaire	
Item	F1	F2	F1	F2
1	0.639		0.419	
2	0.566		0.722	
3	0.554		0.718	
4	0.707		0.664	
5	0.637		0.674	
6	0.619		0.582	
7		0.528		0.683
8		0.666		0.483
9		0.614		0.617
10		0.626		0.516
11		0.414		0.554
12	0.534		0.776	
13	0.470		0.472	
14	0.405		0.767	
15		0.414	0.516	
16		0.434	0.540	
Variance explained (%)	21.2	16.5	29.0	12.3
Cumulative variance (%)	21.2	37.7	29.0	41.3
KMO	0.883		0.887	
Bartlett’s test χ^2^ (*p*)	1332.423 (<0.001)		1193.473 (<0.001)	
Cronbach’s α (overall)	0.873		0.876	
Cronbach’s α (subscale)	0.847	0.762	0.886	0.724
No. of items	9	7	11	5

F1 = Outcome Expectations; F2 = Surgical Process Apprehension. Factor loadings < 0.40 are suppressed.

**Table 4:** Item-level comparison of patient and dentist questionnaire responses.

Item	Patient (*n* = 250)		Dentist (*n* = 200)		*Z*	*p*	FDR-*p*	Cliff’s δ
	Mean ± SD	Median (IQR)	Mean ± SD	Median (IQR)				
M1	3.43 ± 0.97	4 (1)	3.33 ± 0.92	4 (2)	−1.143	0.253	0.280	0.058
M2	3.93 ± 0.72	4 (0)	3.54 ± 0.99	4 (1)	−4.262	<0.001	<0.001	0.209
M3	3.45 ± 0.78	3 (1)	3.15 ± 1.05	3 (2)	−3.036	0.002	0.004	0.158
M4	3.53 ± 0.91	4 (1)	3.76 ± 0.90	4 (1)	−2.770	0.006	0.008	−0.140
M5	3.44 ± 0.94	4 (1)	3.75 ± 0.83	4 (1)	−3.608	<0.001	0.001	−0.181
M6	3.85 ± 0.69	4 (0)	3.83 ± 0.77	4 (0)	−0.082	0.935	0.935	−0.004
M7	2.99 ± 1.00	3 (2)	2.22 ± 0.87	2 (1)	−8.371	<0.001	<0.001	0.437
M8	3.23 ± 0.95	3 (1)	3.02 ± 0.98	3 (2)	−2.526	0.012	0.016	0.132
M9	3.13 ± 0.98	3 (2)	2.66 ± 0.86	2 (1)	−5.426	<0.001	<0.001	0.282
M10	3.46 ± 0.80	4 (1)	3.30 ± 0.84	3 (1)	−2.025	0.043	0.049	0.102
M11	2.91 ± 1.06	3 (2)	2.28 ± 0.92	2 (1)	−6.524	<0.001	<0.001	0.338
M12	3.36 ± 0.87	4 (1)	3.60 ± 1.08	4 (1)	−2.969	0.003	0.005	−0.154
M13	3.50 ± 0.80	4 (1)	3.64 ± 0.86	4 (1)	−2.233	0.026	0.034	−0.108
M14	2.98 ± 0.98	3 (2)	3.34 ± 1.09	4 (2)	−3.652	<0.001	0.001	−0.191
M15	3.56 ± 0.82	4 (1)	3.52 ± 0.90	4 (1)	−0.305	0.760	0.798	0.015
M16	2.42 ± 1.04	2 (1)	3.75 ± 0.78	4 (0.5)	−12.331	<0.001	<0.001	−0.645

IQR, interquartile range; *Z*, Mann–Whitney *U* test statistic; FDR-*p*, Benjamini–Hochberg-adjusted *p*-value; Cliff’s δ, effect size (negligible |δ| < 0.147; small 0.147–0.33; medium 0.33–0.474; large ≥ 0.474). Positive δ indicates higher mean scores in patients; negative δ indicates higher mean scores in dentists. M11 in the patient version was reverse-coded before analysis. Full item content is provided in Table 1. Item-level Cliff’s δ values with 95% confidence intervals are visualized in Supplementary Figure S2, illustrating the bidirectional and domain-specific pattern of between-group differences.

**Table 5:** Comparison of total questionnaire scores between patient and dentist groups.

	Patient (*n* = 250)		Dentist (*n* = 200)		*Z*	*p*	FDR-*p*	Cliff’s δ
	Mean ± SD	Median (IQR)	Mean ± SD	Median (IQR)				
Total scale	53.16 ± 8.44	54 (11)	52.65 ± 8.71	53.5 (13.5)	−0.782	0.434	0.435	0.043

Total scale scores were computed as the sum of the 16 items, with a range of 16–80. Mean absolute gap across items = 0.36. *Z*, Mann–Whitney *U* test statistic; FDR-*p*, Benjamini–Hochberg-adjusted *p*-value; Cliff’s δ, effect size.

**Table 6:** Within-group subgroup analyses of patient and dentist questionnaire scores by demographic and professional characteristics.

Variable	Subgroup	Total Scale	F1	F2	Gap Score
Patient-Age	≤25 (*n* = 37)	51.24 ± 8.21	30.14 ± 5.36	21.11 ± 4.36	1.06 ± 0.41
	26–34 (*n* = 43)	51.44 ± 9.16	30.33 ± 5.34	21.12 ± 4.71	1.16 ± 0.40
	35–44 (*n* = 49)	50.88 ± 9.38	30.04 ± 5.56	20.84 ± 5.00	1.10 ± 0.34
	45–54 (*n* = 52)	55.52 ± 6.58	33.29 ± 4.35	22.23 ± 3.12	1.10 ± 0.39
	≥55 (*n* = 69)	55.10 ± 7.97	32.51 ± 4.71	22.59 ± 4.07	0.98 ± 0.31
	*p*	0.002	<0.001	0.120	0.250
Patient-Sex	Female (*n* = 132)	51.68 ± 8.65	30.78 ± 5.35	20.90 ± 4.35	1.09 ± 0.37
	Male (*n* = 118)	54.81 ± 7.93	32.22 ± 4.87	22.59 ± 4.04	1.04 ± 0.37
	*p*	<0.001	0.022	0.002	0.324
Patient-Education	Primary/middle (*n* = 35)	58.69 ± 6.48	34.26 ± 4.00	24.43 ± 3.34	0.96 ± 0.36
	High school (*n* = 47)	56.43 ± 6.96	33.17 ± 4.49	23.26 ± 3.27	1.09 ± 0.46
	Bachelor’s (*n* = 117)	51.45 ± 8.65	30.56 ± 5.31	20.89 ± 4.46	1.07 ± 0.37
	Postgraduate (*n* = 51)	50.27 ± 7.87	30.02 ± 5.10	20.25 ± 4.09	1.10 ± 0.30
	*p*	<0.001	<0.001	<0.001	0.449
Dentist-Age	≤25 (*n* = 40)	58.00 ± 5.11	43.63 ± 4.05	14.38 ± 3.11	0.99 ± 0.37
	26–34 (*n* = 62)	56.74 ± 8.94	43.10 ± 6.74	13.65 ± 3.92	1.11 ± 0.38
	35–44 (*n* = 39)	48.18 ± 6.83	35.51 ± 5.29	12.67 ± 2.12	1.00 ± 0.36
	45–54 (*n* = 30)	46.43 ± 6.36	33.50 ± 4.99	12.93 ± 2.10	1.16 ± 0.30
	≥55 (*n* = 29)	48.93 ± 7.82	35.52 ± 5.70	13.41 ± 2.78	1.11 ± 0.39
	*p*	<0.001	<0.001	0.184	0.286
Dentist-Sex	Female (*n* = 111)	52.82 ± 7.99	39.69 ± 6.80	13.13 ± 3.02	1.06 ± 0.33
	Male (*n* = 89)	52.43 ± 9.57	38.55 ± 7.22	13.88 ± 3.14	1.09 ± 0.41
	*p*	0.762	0.262	0.106	0.622
Dentist-Experience	0–5 yr (*n* = 76)	57.47 ± 6.15	43.59 ± 4.80	13.88 ± 3.38	1.07 ± 0.39
	6–10 yr (*n* = 53)	51.15 ± 10.23	38.06 ± 7.84	13.09 ± 3.40	1.04 ± 0.36
	≥11 yr (*n* = 71)	48.59 ± 7.31	35.31 ± 5.60	13.28 ± 2.44	1.09 ± 0.35
	*p*	<0.001	<0.001	0.341	0.674
Dentist-Specialty	General (*n* = 52)	56.48 ± 6.19	42.12 ± 4.78	14.37 ± 3.51	1.08 ± 0.39
	Periodontology (*n* = 42)	46.43 ± 6.81	34.24 ± 5.85	12.19 ± 1.89	1.08 ± 0.37
	OMFS (*n* = 39)	56.54 ± 8.55	42.31 ± 6.28	14.23 ± 3.59	1.09 ± 0.34
	Prosthodontics (*n* = 38)	47.16 ± 7.45	34.71 ± 6.35	12.45 ± 2.01	1.07 ± 0.35
	Other (*n* = 29)	56.72 ± 7.99	42.76 ± 6.51	13.97 ± 3.28	1.02 ± 0.39
	*p*	<0.001	<0.001	<0.001	0.781

Values are mean ± SD. *p*-values were obtained from the Kruskal–Wallis test (≥3 groups) or the Mann–Whitney *U* test (2 groups); an independent-samples *t*-test was used where normality and homogeneity-of-variance assumptions were satisfied. Pairwise post hoc contrasts (Bonferroni-adjusted) are detailed in the main text.

## Data Availability

The raw data supporting the conclusions of this article will be made available by the authors on request.

## Supplementary Materials

The following supporting information can be downloaded at: https://www.mdpi.com/article/10.3390/healthcare14152268/s1. Table S1: STROBE checklist for cross-sectional studies; Figure S1: Scree plot of eigenvalues from exploratory factor analysis for the patient and dentist samples; Figure S2: Forest plot of item-level Cliff’s δ values with 95% confidence intervals for patient–dentist comparisons across the 16 questionnaire items.

## Abbreviations

The following abbreviations are used in this manuscript:

CVI	Content Validity Index
FDR	False Discovery Rate
IQR	Interquartile Range
KMO	Kaiser–Meyer–Olkin
OMFS	Oral and Maxillofacial Surgery
PAF	Principal Axis Factoring
PROMs	Patient-Reported Outcome Measures
S-CVI/Ave	Scale-level Content Validity Index
SD	Standard Deviation
SPSS	Statistical Package for the Social Sciences
STROBE	Strengthening the Reporting of Observational Studies in Epidemiology.
