# Supplementary material for: Patient Expectations and Dentists’ Perceptions of Those Expectations in Dental Implant Therapy: A Mirror-Design Study
Source: Healthcare (Basel). 2026 Jul 24;14(15):2268. doi: 10.3390/healthcare14152268 (PMC13465889; doi:10.3390/healthcare14152268)
Supplement: Supplementary file 1 [file healthcare-14-02268-s001.zip › healthcare-4373365-supplementary.pdf]

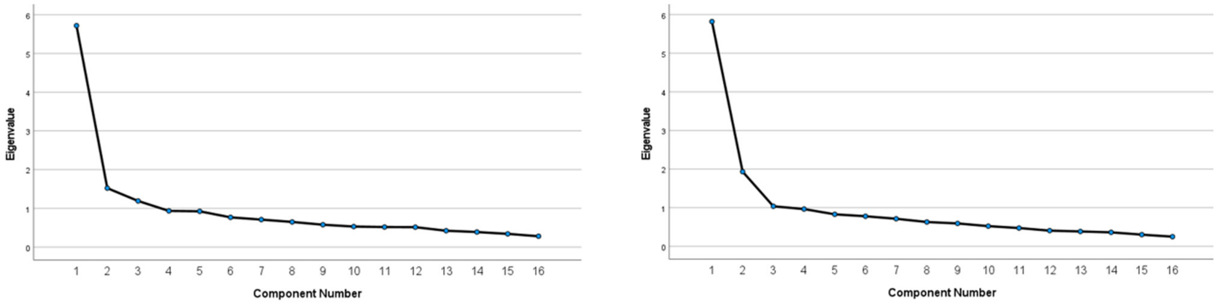

Figure S1. Scree plot of eigenvalues from exploratory factor analysis for the patient and dentist samples, supporting a two-factor solution.

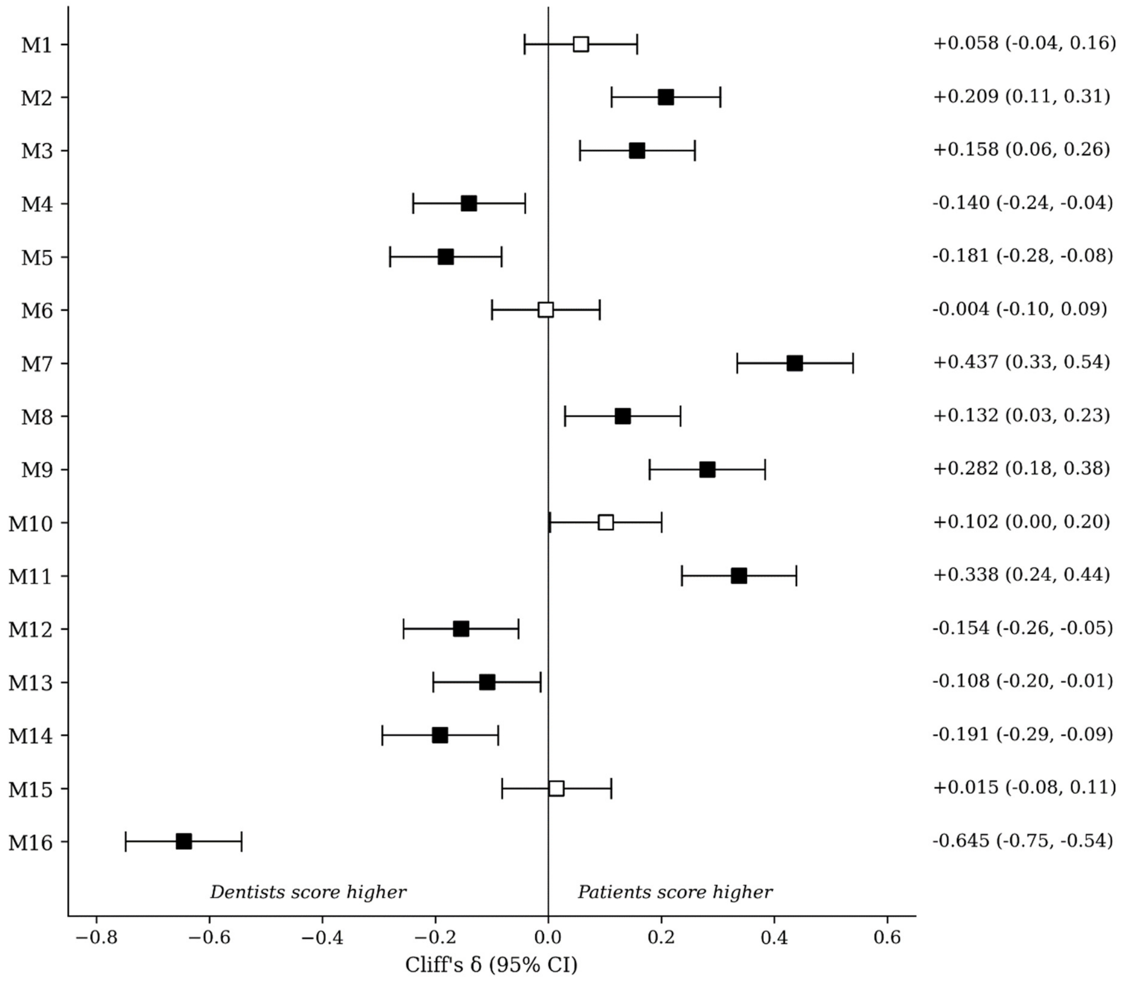

Figure S2. Forest plot of item-level Cliff's δ values with 95% confidence intervals for patient–dentist comparisons across the 16 questionnaire items (M1–M16). Filled squares indicate items reaching statistical significance after Benjamini–Hochberg correction (FDR-p < 0.05); open squares indicate non-significant items (M1, M6, M10, M15). Positive δ values indicate higher mean scores in patients; negative δ values indicate higher mean scores in dentists. † Cross-loaded items: M15 and M16 loaded onto F2 in the patient sample but onto F1 in the dentist sample (see Table 2). Full item content is provided in Supplementary Material S1.

Supplementary Table S1. STROBE Statement - Checklist of items that should be included in reports of ***cross-sectional studies***

	Item No	Recommendation	
**Title and abstract**	1	(*a*) Indicate the study's design with a commonly used term in the title or the abstract	Title p.1 / Abstract p.1
		(*b*) Provide in the abstract an informative and balanced summary of what was done and what was found	Abstract p.1
		**Introduction**	
Background/rationale	2	Explain the scientific background and rationale for the investigation being reported	Introduction pp.1-2; paragraphs 1-4
Objectives	3	State specific objectives, including any prespecified hypotheses	Introduction pp.2-3; final paragraph
		**Methods**	
Study design	4	Present key elements of study design early in the paper	Section 2.1 (p.3)
Setting	5	Describe the setting, locations, and relevant dates, including periods of recruitment, exposure, follow-up, and data collection	Section 2.1, 2.2 (p.3)
Participants	6	(*a*) Give the eligibility criteria, and the sources and methods of selection of participants	Section 2.2 (p.3)
Variables	7	Clearly define all outcomes, exposures, predictors, potential confounders, and effect modifiers. Give diagnostic criteria, if applicable	Section 2.4, 2.6 (pp.3-4-5)
Data sources/ measurement	8*	For each variable of interest, give sources of data and details of methods of assessment (measurement). Describe comparability of assessment methods if there is more than one group	Section 2.4, 2.5 (pp.3-4)
Bias	9	Describe any efforts to address potential sources of bias	Section 2.2, 2.5 (pp.3-4-5), Discussion (Limitations)
Study size	10	Explain how the study size was arrived at	Section 2.3 (p.3)
Quantitative variables	11	Explain how quantitative variables were handled in the analyses. If applicable, describe which groupings were chosen and why	Section 2.6 (pp.4-5)
Statistical methods	12	(*a*) Describe all statistical methods, including those used to control for confounding	Section 2.6 (pp.4-5)
		(*b*) Describe any methods used to examine subgroups and interactions	Section 2.6 (pp.4-5); Section 3.5
		(*c*) Explain how missing data were addressed	Section 2.5 (p.4)
		(*d*) If applicable, describe analytical methods taking account of sampling strategy	Consecutive sampling
		(*e*) Describe any sensitivity analyses	N/A
		**Results**	
Participants	13*	(a) Report numbers of individuals at each stage of study—eg numbers potentially eligible, examined for eligibility, confirmed eligible, included in the study, completing follow-up, and analysed	Section 2.2 (p.3); Section 3.1 (p.5)
		(b) Give reasons for non-participation at each stage	Section 2.2 (p.3)
		(c) Consider use of a flow diagram	Reported in text (Section 2.2)
Descriptive data	14*	(a) Give characteristics of study participants (eg demographic, clinical, social) and information on exposures and potential confounders	Section 3.1; Table 1 (pp. 5–6)
		(b) Indicate number of participants with missing data for each variable of interest	Section 2.5 (p. 4) no missing data
Outcome data	15*	Report numbers of outcome events or summary measures	Section 3.2-3.5; Tables 2-5 (pp.6-13)
Main results	16	(*a*) Give unadjusted estimates and, if applicable, confounder-adjusted estimates and their precision (eg, 95% confidence interval). Make clear which confounders were adjusted for and why they were included	Section 3.3-3.5; Table 3-5 (pp.7-13)
		(*b*) Report category boundaries when continuous variables were categorized	Table 1 (pp.5-6)
		(*c*) If relevant, consider translating estimates of relative risk into absolute risk for a meaningful time period	N/A
Other analyses	17	Report other analyses done—eg analyses of subgroups and interactions, and sensitivity analyses	Section 3.5 (Subgroup Analyses); Section 3.5.3 (Spearman correlations)
		**Discussion**	

Key results	18	Summarise key results with reference to study objectives	Discussion paragraph 1 (pp.11-12)
Limitations	19	Discuss limitations of the study, taking into account sources of potential bias or imprecision. Discuss both direction and magnitude of any potential bias	Discussion paragraph 8 (p.14)
Interpretation	20	Give a cautious overall interpretation of results considering objectives, limitations, multiplicity of analyses, results from similar studies, and other relevant evidence	Discussion, paragraphs 2–7 (pp. 12–14)
Generalisability	21	Discuss the generalisability (external validity) of the study results	Discussion, paragraph 8 (p. 14)
**Other information**			
Funding	22	Give the source of funding and the role of the funders for the present study and, if applicable, for the original study on which the present article is based	Funding statement (end of manuscript)

*Give information separately for exposed and unexposed groups.

Note: An Explanation and Elaboration article discusses each checklist item and gives methodological background and published examples of transparent reporting. The STROBE checklist is best used in conjunction with this article (freely available on the Web sites of PLoS Medicine at http://www.plosmedicine.org/, Annals of Internal Medicine at http://www.annals.org/, and Epidemiology at http://www.epidem.com/). Information on the STROBE Initiative is available at www.strobe-statement.org.
